# Revisiting nocturnal heart rate and heart rate variability in insomnia: A polysomnography‐based comparison of young self‐reported good and poor sleepers

**DOI:** 10.1111/jsr.13278

**Published:** 2021-02-23

**Authors:** Jan Cosgrave, Jessica Phillips, Ross Haines, Russell G. Foster, David Steinsaltz, Katharina Wulff

**Affiliations:** ^1^ Sleep and Circadian Neuroscience Institute NDCN The Sir William Dunn School of Pathology University of Oxford Oxford UK; ^2^ Department of Clinical, Educational and Health Psychology University College London London UK; ^3^ Department of Statistics University of Oxford Oxford UK; ^4^ Departments of Radiation Sciences and Molecular Biology Umeå University Umeå Sweden; ^5^ Wallenberg Centre for Molecular Medicine (WCMM) Umeå University Umeå Sweden

**Keywords:** heart rate variability, hyperarousal, insomnia, nocturnal heart rate, polysomnography, sympathetic nervous system

## Abstract

Primary insomnia is often considered a disorder of 24‐hr hyperarousal. Numerous attempts have been made to investigate nocturnal heart rate (HR) and its variability (HRV) as potential pathophysiological hallmarks of altered arousal levels in insomnia, with mixed results. We have aimed to overcome some of the pitfalls of previous studies by using a young, medication‐free, age‐ and gender‐matched population consisting of 43 students aged 18–30 years half with a subthreshold insomnia complaint. We employed at‐home ambulatory polysomnography and compared this attenuated insomnia group to a good sleeping group. The poor sleepers had significantly higher wake after sleep onset, arousal count, mean HR in all sleep stages (with the exception of Stage 1) and lower sleep efficiency. Consistent with previous research, we also found a significant group‐by‐sleep stage interaction in the prediction of nocturnal HR, highlighting the insomnia group to have a lower wake–sleep HR reduction compared to good sleepers. When restricting our analyses to insomnia with objectively determined short sleep duration, we found significantly lower standard deviation of RR intervals (SDNN; a measure of HRV) compared to good sleepers. Taken together, this lends credence to the hyperarousal model of insomnia and may at least partially explain the increased prevalence of cardiovascular morbidity and mortality observed in patients with insomnia.

## 1 INTRODUCTION

Chronic insomnia has been reported to be associated with several indices of elevated autonomic activity, including increased metabolic rate, body temperature, activation of the hypothalamic–pituitary–adrenal axis activity, brain glucose metabolism and reduced heart rate variability (HRV) during sleep (Morin & Benca, 2012; Morin & Jarrin, 2013; Riemann et al., 2010). Cumulatively, this would indicate insomnia to be at least partially pathophysiologically driven by mechanisms of altered activation of the autonomic nervous system (ANS; Sateia & Nowell, 2004). This has informed the development of the hyperarousal theory of insomnia (Riemann et al., 2010).

Of these metrics, HRV has gained considerable attention, in part as it is seen across the diagnostic spectrum, including in disorders that are heavily comorbid with insomnia (e.g. major depressive disorder; Eddie et al., 2020), and because heart rate (HR) itself is dually modulated by both sympathetic and parasympathetic branches of the ANS (Malik & Camm, 1993). Usually denoted by the variance in the inter‐beat interval or distance between two consecutive R waves, referred to as the R–R interval, HRV is consequently often considered a biomarker of ANS regulatory processes (Beissner et al., 2013; McCraty & Shaffer, 2015). Therefore, if HRV were found to be different in insomnia, it would signify increased physiological hyperarousal and inform the pathogenesis of the disorder (Riemann et al., 2010).

Generally, high HRV is considered to reflect a healthy cardiovascular autonomic function. A low nocturnal HRV is a sign of decreased cardiovascular adaptability, and consequently it is associated with higher risk of initiation and progression of cardiovascular disorders (Jarrin et al., 2016).

However, the literature on HR and HRV in insomnia is mixed. Some studies indicate attenuated HRV in both waking and resting conditions (Bonnet & Arand, 1997; Cellini et al., 2014; Spiegelhalder et al., 2011). Others report no significant differences (Eddie et al., 2020; Malik & Camm, 1993). A systematic review by Dodds et al. (2016) provided an overview of 22 studies comparing HRV between patients with insomnia and controls, and concluded that it was not possible to confirm that HRV is “reliably impaired in insomnia patients”, due to the high risk of bias in a majority of the studies reviewed, as well as the lack of consistency in the HRV findings in patients with insomnia (Dodds et al., 2016).

The authors noted several issues with the current literature, such as the use of laboratory settings (known to impact sleep in insomnia), the influence of arousals on HRV, and the prevalence of medication and comorbid medical disorders in the samples (Dodds et al., 2016).

### 1.1 The present study

To help clarify this, we wished to revisit nocturnal HR and HRV in insomnia, whilst addressing the issues reported by Dodds et al., (2016). First, we conducted ambulatory or at‐home polysomnography (PSG) alongside electrocardiography (ECG). Second, we selected “good sleepers” from the same population as the “poor sleeping” group, matching for age and gender. We chose a younger undiagnosed student sample (aged 18–30 years) not recruited from a sleep centre, thereby representing an attenuated sample and reducing confounding from medication or comorbid medical disorders comparative to previous studies. Furthermore, we excluded any participant taking psychotropic medication or any medication known to impact sleep. Taken together, these measures were designed to narrow the manipulation to the subjective reporting of sleep between groups. As arousals are frequently allied or potentially induced by cardiac fluctuations throughout the night (Bonnet & Arand, 1997), they were excluded from the HR and HRV analysis. To explore the impact of arousals separately, the arousal counts (partitioned by sleep stage) across the sleep period for each participant were also calculated and compared across groups.

We hypothesised that the insomnia group would display (i) objectively poorer sleep based on ambulatory PSG (primarily in sleep efficiency and sleep‐onset latency, in line with Spiegelhalder et al., (2015)); (ii) a greater number of arousals and heightened arousal index across the night; (iii) increased nocturnal HR and decreased HRV across different stages of sleep throughout the night. Furthermore, we were attempting (iv) to replicate the findings of Spiegelhalder et al., (2011) with a significant group‐by‐sleep stage interaction for both nocturnal HR and HRV as the outcome variables. Finally, as an exploratory analysis, we wished to examine the impact of insomnia with short sleep duration on HRV (v).

## 2 METHODOLOGY

### 2.1 Participants and recruitment

The sample consisted of 47 university students aged 18–30 years from Oxford. The final sample included 43 students, 23 in the insomnia group (mean [*SD*] age 23.7 [3.5] years, 14 women), and 20 good sleepers (mean [*SD*] age 22.7 [3.2] years, 11 women), with four students excluded from the analysis. Reasons for exclusion were discontinuation prior to PSG set‐up (two), non‐compliance with the protocol (one), and a suspected circadian rhythm disorder (one). For the ECG analyses, a further participant was excluded due to the detection of a cardiac arrhythmia. This subject was included for the PSG group comparisons.

Students were recruited via poster advertisements and email. The study protocol was approved by the National Research Ethics Service (NRES) Committee North West‐Liverpool Central (Research Ethics Committee [REC]: 14/NW/1142) and all participants gave written informed consent.

### 2.2 Eligibility and selection

Eligibility for the study was based upon subjective reporting of sleep quality and insomnia using the Pittsburgh Sleep Quality Index (PSQI) and the Insomnia Severity Index (ISI). The standardised cut‐off score for poor quality sleep in the PSQI is 5. The ISI is a brief questionnaire devised to measure both the night‐time and day‐time elements of insomnia. The ISI ranges from 0 to 28, with scores of ≥10 considered optimal for detecting insomnia in community samples (Morin et al., 2011). Both measures have shown good psychometric properties for use in both patients and healthy controls (Backhaus et al., 2002; Carpenter & Andrykowski, 1998; Morin et al., 2011).

For this study, “good sleepers” were required to have a Pittsburgh Sleep Quality Index (PSQI) score of ≤3 and an ISI score of ≤6. The insomnia group were required to have a PSQI of ≥8 (3 points higher than the standard cut‐off score of 5) and an ISI score of ≥10, creating a degree of separation in the subjective reporting of sleep quality between the groups. Exclusion criteria included a diagnosis of a psychotic disorder (past or present), taking medication known to impact sleep, taking any psychotropic medication, brain injury, epilepsy, shift work, hospitalisation in the previous 6 months, and travelling through two or more time zones in the previous 2 weeks. Depression and anxiety scores were recorded using the Depression and Anxiety Stress Scale (21‐item version; Henry & Crawford, 2005).

### 2.3 Ambulatory polysomnography (PSG)

All participants underwent 2 consecutive nights of ambulatory PSG sleep monitoring. The first night served as an adaptation night. The second night was used for the present analyses. Both nights were set up in the participants’ home environment. Recordings were set to run 14 hr with the start time determined by the participant’s estimation of the earliest possible time they could go to bed. Participants were asked to be extra vigilant when completing their sleep diaries to determine lights off and lights on. Where these appeared discrepant from the PSG recording, we triangulated information from their estimation of when they would go to bed that night and when the ECG recordings began (as participants were instructed to apply these when they got into bed). Where there was doubt, the objective data were given priority so as not to unduly inflate time in bed (TIB) estimates, which could bias group effects.

The montage included 19 electrodes mounted bilaterally: 10 on the scalp according to the international 10–20 system: Fp1, Fp2, F3, F4, C3, C4, P3, P4, O1, O2, as well as A1 and A2 (for offline referencing), two electro‐oculography (EOG, for eye movements), three electromyography (EMGs, for musculus mentalis movements), ground (at FPz) and a reference electrode at position FCz. The Somnomedics signal was recorded with a 24‐channel Somnoscreen plus electroencephalography (EEG) amplifier (Somnomedics Inc.); all electrodes were referenced to contralateral mastoids and digitised with 128 Hz sampling rate.

The EEG recordings were manually scored by an experienced rater at 30‐s epochs according to the criteria of the American Academy of Sleep Medicine (AASM) Manual (Berry et al., 2012), with the addition of Stage 4 scoring to highlight >50% of slow waves present in a 30‐s epoch. This was added to explore if there were more pronounced differences between groups with increased delta activity (as exploration of deep sleep had not been possible in previous studies; Spiegelhalder et al., (2011)). Five recordings (12%) were second scored with a 90% concordance rate to ensure a high standard of both scoring and inter‐rater reliability. All arousals were manually scored by visual inspection following the AASM guidelines. An arousal was defined as an abrupt shift of EEG frequency including alpha, theta and/or frequencies >16 Hz (but not spindles), lasting at least 3 s in duration and preceded by at least 10 s of stable sleep (Berry et al., 2012).

Sleep recordings were evaluated for the following parameters of sleep continuity: *total sleep time* (TST), defined as time between sleep onset and final awakening, including arousals but excluding periods defined as movement or awake; *sleep efficiency*, ratio of TST to TIB × 100%; *wake after sleep onset* (WASO), difference between TST and sleep period time, time from sleep onset until final awakening, including movement and time awake; *sleep‐onset latency*, time from lights out until sleep onset, defined as first epoch of Stage 1; *arousal count*; *arousal index*, number of arousals per hour of TST (Sleep Disorders Atlas Task Force of the American Sleep Disorders Association, 1992). Sleep architecture parameters were amounts of Stages 1, 2 and slow‐wave sleep marked as either Stages 3 or 4, rapid eye movement sleep (REM) as a duration and percentage of TST and REM latency (the amount of time taken before the first bout of REM sleep).

### 2.4 ECG recording and analysis

During PSG recordings, the HR signal was obtained by two surface disposable electrodes that were placed just below the right clavicle and on the anterior axillary line on the costal arch. Participants were instructed to do this before getting into bed. The ECG sampling rate was 256 Hz. All epochs were inspected visually for artefacts or inaccurate detections. Arousals during the EEG recording often co‐occur with fluctuations in HR. As arousals tend to be more prevalent in insomnia sufferers, five consecutive heartbeats of ECG recording pre‐ and post‐arousal were removed to prevent arousals biasing the HR signal. The HR throughout the recording was determined using timestamps of R within the QRS complex (three deflections corresponding to the depolarisation of the right and left ventricles) from lights off to lights on. Furthermore, all epochs of wake, arousals or artefacts were discarded from analysis. For each epoch, the mean HR (beats /min [bpm]) was calculated from the R–R interval (RRI, distance between two consecutive R waves) time series.

The HRV is generally derived from the analysis of inter‐beat timings, which are referred to as the RRI. Descriptive measures of the RRI such as the standard deviation (SDNN) or the square root of the mean of the sum of the squares of differences between adjacent RRIs (rMSSD) over certain time epochs are examples of time‐domain measures used in the examination of HRV (Dodds et al., 2016). For evaluating HRV in this study, both time‐ and frequency‐domain methods were applied. In the time domain, the SDNNs were analysed for each epoch. The SDNN reflects overall HRV. Frequency‐domain measures based on the fast‐Fourier transform (FFT) algorithm were applied on the regularly sampled interpolation of the RRI time series (10.24 Hz). Spectral power was calculated for three frequency bands: low frequency (LF; 0.04–0.15 Hz), high frequency (HF; 0.15–0.4 Hz), and total power (0.04–0.4 Hz). The HF was selected as the frequency‐domain measure for this study, which is now considered a marker for vagal tone (Laborde et al., 2017). Generally, a higher vagal tone is seen as “adaptive” reflecting improved cognitive performance, alongside better emotional and health regulation (Thayer et al., 2009).

The HR and SDNN were computed across 30‐s epochs (in line with the sleep staging), whereas HF was computed in 5‐min bins (in line with the measurement standards recommended by Laborde et al., (2017)). To determine the sleep stage associated with each 5‐min epoch, the most commonly scored stage across the 10 epochs was used. Where it was not clear which was the dominant sleep stage for the 5‐min epoch (e.g. if five epochs were Stage 2 and the other five were REM), the bin was excluded from modelling analyses. This resulted in 115 5‐min bins being excluded from the analysis across all of the subjects.

### 2.5 Statistical analysis

Statistical analyses were performed using the R Software Environment (R Core Team, 2017), with the packages “nlme” (for mixed‐effects models; Pinheiro et al., 2017); “tidyverse” (for data wrangling and visualisation; Wickham et al., 2019); “chron” (for handling of time data; James & Hornik, 2017); “Hmisc” and “gridExtra” (for visualising results; Harrell and Dupont (2006) & Auguie et al. (2017)).

All measures were examined for their distributional properties. Where measures had skewed distributions, the Wilcoxon rank‐sum test with continuity correction was employed. For these skewed measures, the median (with *SD*s in brackets) and the Wilcoxon test statistic were reported. The *p* values were corrected for multiple testing using the Benjamini and Hochberg (1995) correction method, which controls for false discovery rate, as opposed to the more commonly employed Bonferroni method, which controls for the family‐wise error rate.

To explore the impact of demographic (e.g. age, gender) and sleep (e.g. group and sleep stage) variables on nocturnal HR, SDNN and HF, we used mixed‐effects statistical models with a random effect for the individual and a group × sleep stage interaction (consistent with previous research).

## 3 RESULTS

### 3.1 Demographics and polysomnography

The difference in the mean age between the two groups was not statistically significant (*t* = 1.078; *p* = .287). The mean (*SD*) subjectively reported sleep quality (PSQI) in the good sleepers group was 2.3 (0.9), as opposed to 10.1 (2.2) in the insomnia group. The mean (*SD*) insomnia scores (ISI) were 14.4 (3.3) for the insomnia group and 1.5 (1.5) for the good sleepers, highlighting a sizeable difference in subjective sleep quality and insomnia between groups. Group summary statistics are presented in Table 1.

**TABLE 1:** Demographic overview of the good sleepers (*n* = 19) and insomnia group (*n* = 23)

Demographic variable	Good sleepers	Insomnia group	*W*	*p*	*p*_adj_.
	Mean (*SD*)	Mean (*SD*)			
Gender	11 women	14 women	–	–	–
Age, years	22.7 (3.2)	23.7 (3.5)	–	–	–
PSQI score	2.3 (0.9)	10.1 (2.2)	–	–	–
ISI score	1.5 (1.5)	14.4 (3.3)	–	–	–
Depression score	4.0 (5.1)	14.0 (9.9)	75.5	<.001	<.001
Anxiety score	4.0 (2.6)	8.0 (7.6)	82.5	<.001	<.001

ISI, Insomnia Severity Index; PSQI, Pittsburgh Sleep Quality Index; *SD*, standard deviation.

The ‘adjusted *p* values’ throughout this paper were corrected for multiple testing by the Benjamini–Hochberg correction (Benjamini & Hochberg, 1995), which controls for false discovery rate. The well‐known Bonferroni method controls for the family‐wise error rate. The adjusted *p* values are reported in the *p*adj. column.

The PSG data show that the insomnia group had significantly lower sleep efficiency and significantly longer WASO, after correcting for multiple comparisons (Table 2).

**TABLE 2:** Polysomnography (PSG) parameters of the insomnia and good sleeping groups (*n* = 42)

Measurement	Good sleepers	Insomnia group	Group differences		
	Mean (*SD*)	Mean (*SD*)	*t*	*p*	*p*_adj_
Sleep efficiency, %	94.55 (4.2)	88.43 (7.1)	3.46	**.002**	**.012**
TIB, hr	7:57 (0:48)	8:43 (1:08)	−2.59	**.013**	.052
TST, hr	7:30 (0:42)	7:41 (1:08)	−0.66	.512	.683
SOL, hr	0:14:(0:24)	0:36 (0:32)	−2.46	**.018**	.054
REM latency, hr	1:26 (0:44)	1:30 (0:36)	−0.35	.733	.733
WASO, hr	0:27 (0:25)	1:02 (0:40)	−3.49	**.001**	**.012**
REM, hr	1:52 (0:33)	1:43 (0:33)	0.83	.411	.616
Stage 1, hr	0:23 (0:14)	0:21 (0:15)	0.40	.695	.733
Stage 2, hr	3:42 (0:33)	3:56 (0:51)	−1.07	.291	.499
Stage 3, hr	0:24 (0:10)	0:30 (0:09)	−1.79	.081	.194
Stage 4, hr	1:09 (0:21)	1:11 (0:19)	−0.35	.726	.733
Arousal Index	8.70 (2.20)	9.87 (4.27)	−1.10	.258	.499

REM, rapid eye movement sleep; *SD*, standard deviation; SOL, sleep‐onset latency; TIB, time in bed; TST, total sleep time; WASO, wake after sleep onset.

*p* values reported here were corrected for multiple testing using the Benjamini and Hochberg correction method (Benjamini & Hochberg, 1995), which controls for false discovery rate, as opposed to the more commonly employed Bonferroni method, which controls for the family‐wise error rate. *p* values below the significance threshold of .05 are in bold.

### 3.2 Arousals

**FIGURE 1:**
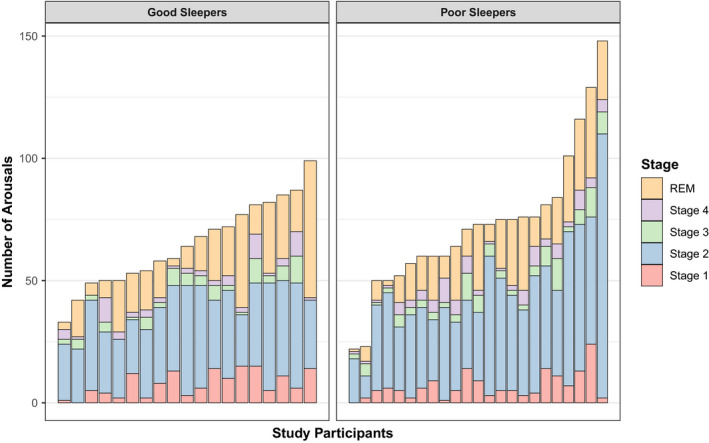
Inter‐individual variability in arousal counts across sleep architecture for good and poor sleepers. Each bar represents an individual participant. Good and poor sleepers are on the left‐ and right‐hand sides of the graph, respectively. The highest scorers belong to the poor sleeping group, although clear group differences do not appear to be present. A chi‐square test of independence showed the poor sleepers differ significantly in the way the arousals are distributed across the stages of sleep compared to the good sleeping group, highlighting a group and stage association with respect to arousal count

For the total arousal count by sleep stage (Table 3), a chi‐square test of independence showed significant between‐group differences in the way the arousals are distributed across the sleep stages (χ^2^(4), *n* = 42) = 16.0, *p* = .003), highlighting a group and stage association with respect to arousal count. Vast inter‐individual differences were found in the number of arousals across each sleep stage and across the night (Figure 1). There was no significant difference in arousal index between groups (*t* = −1.10; *p* = .26; Table 2).

**TABLE 3:** Two‐way contingency table with arousal counts across each sleep stage for good sleepers (*n* = 19) and the insomnia group (*n* = 23)

Sleep stage	Good sleepers		Insomnia group	
	Count	Mean (*SD*)	Count	Mean (*SD*)
Stage 1	146	8.1 (4.9)	155	7.1 (5.4)
Stage 2	607	31.9 (8.2)	926	40.3 (19.7)
Stage 3	75	4.4 (2.9)	113	4.9 (3.5)
Stage 4	63	3.5 (3.1)	92	4.0 (2.7)
REM	343	18.1 (12.8)	390	17.0 (9.7)

### 3.3 Nocturnal heart rate

**FIGURE 2:**
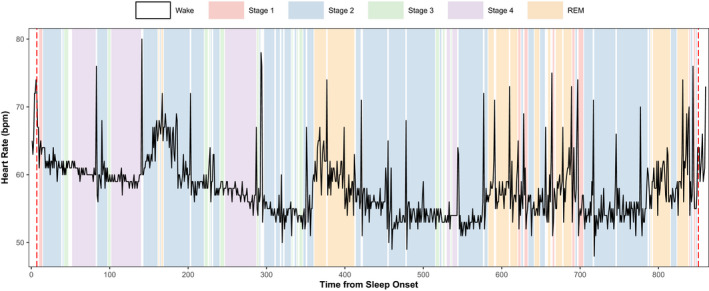
The trajectory of nocturnal heart rate (HR) across the stages of sleep throughout the night. The nocturnal HR of a good sleeper averaged over 30‐s epochs is displayed throughout the sleeping period. Sleep stages are overlaid with different colours to highlight the fluctuations in HR attached to different sleep stages throughout the night. Epochs of Stages 3 and 4 are more frequent and longer in duration during the first half of the night and are generally accompanied with dips in nocturnal HR. This graph also highlights the influence of circadian phase on nocturnal HR with the lowest points of HR occurring the mid‐point of the night emulating a cosine curve (consistent with the nadir of core body temperature; Massin et al., 2000; Nakagawa et al., 1998). Furthermore, heightened HR across epochs of rapid eye movement (REM; found primarily across the second half of the night) is observed, the timing and duration of which are generally considered to be under circadian control (Beersma & Gordijn, 2007)

The HR superimposed on sleep stages across the night revealed a decline in HR from sleep start towards mid‐sleep across Stages 2, 3 and 4, while Stage 1, REM sleep and waking‐up, tend to be associated with an increase in HR (Figure 2).

**FIGURE 3:**
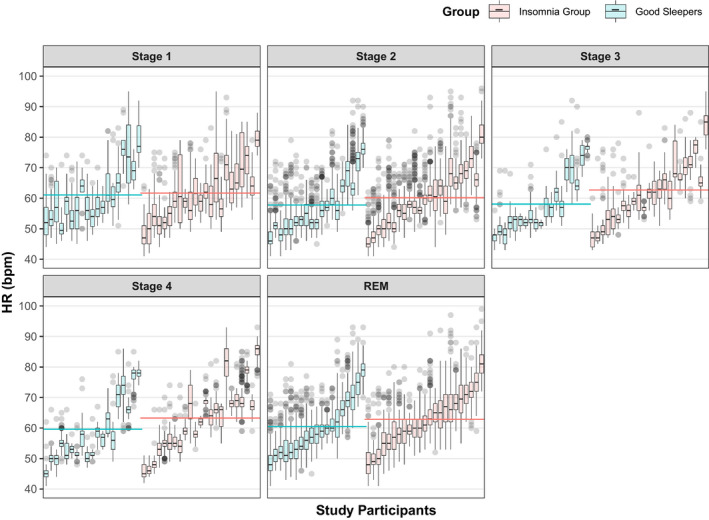
Boxplots highlighting the variability in nocturnal heart rate (HR) between and within individuals across sleep stages (*n* = 42). Each boxplot represents an individual participant and the grey dots represent outliers. The dashed red line indicates mean HR for each group. Green boxes denote individuals in the good sleeping group, while orange shading denotes individuals in the insomnia group. While the mean HR of the two groups is closely matched at sleep Stage 1, the difference becomes increasingly more apparent towards deeper stages of sleep (Table 4). Considerable inter‐individual variability between participants is also demonstrated

The poor sleepers had a significantly higher mean HR across sleep Stages 2, 3, 4 and REM compared to the good sleepers (Table 4; Figure 3). Additionally, the distribution of nocturnal HR within and between individuals for each sleep stage demonstrates considerable inter‐individual variability (Figure 3).

**TABLE 4:** Comparisons of mean nocturnal heart rate (HR) across sleep stages for good and poor sleepers (*n* = 42)

Sleep stage	HR good sleepers, bpm	HR insomnia group, bpm	*t*	*p*	*p*_adj_
Stage 1	60.6	61.2	−1.39	.164	.164
Stage 2	57.2	59.6	−17.7	**<.001**	**<.001**
Stage 3	57.6	62.2	−12.2	**<.001**	**<.001**
Stage 4	59.1	62.8	−13.3	**<.001**	**<.001**
REM	60.0	62.4	−11.8	**<.001**	**<.001**

bpm, beats/min; HR, heart rate.

*p* values below the significance threshold of .05 are in bold.

**FIGURE 4:**
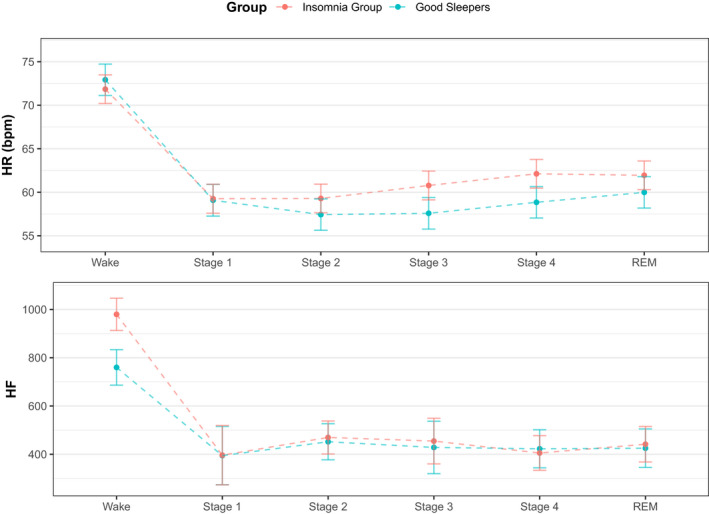
Model estimates of heart rate (HR) and high frequency heart rate variability based on frequency bands from spectral analysis of HR variability (HF). Top: The difference in nocturnal HR across sleep stages and groups is highlighted, with the reduction in HR for the insomnia group attenuated across all stages of sleep (apart from Stage 1) when compared to the good sleepers. Bottom: HF is highlighted across sleep stages and groups. The insomnia group display increased HF ratio during Wake across the night, which narrows from Stages 1 to rapid eye movement (REM). Values are mean ± *SE*

The mixed‐effects models highlighted no significant effect of group (*F*_38,1_ = 0.71, *p* = .41) or age (*F*_38,1_ = 0.83, *p* = .37) on nocturnal HR, but gender (*F*_38,1_ = 8.82, *p* < .01), sleep stage (*F*_42,047,5_ = 3,345.0, *p* < .0001) and the interaction between group and sleep stage (*F*_42,047,5_ = 63.9, *p* < .0001) were significant predictors of nocturnal HR (Table 5). Figure 4 (top) illustrates this interaction, highlighting lower sleep–wake reduction of the insomnia group. Please refer to Table S1 to review the full model output.

**TABLE 5:** Significance testing of mixed‐effects model predictors with nocturnal heart rate (HR), standard deviation of the R–R interval (SDNN) and high frequency heart rate variability based on frequency bands from spectral analysis of HR variability (HF) ratio as primary outcome variables

	*df*_n_	*df*_d_	*F*	*p*
Outcome variable: HR				
Intercept	1	42,047	2,608.3	<.0001
Age	1	38	0.83	.37
Gender	1	38	8.82	**.01**
Group	1	38	0.71	.41
Stage	5	42,047	3,345.0	**<.0001**
Group: Stage	5	42,047	63.90	**<.0001**
Outcome variable: SDNN				
Intercept	1	156	383.84	<.0001
Group	1	40	0.35	.56
Stage	4	156	9.27	**<.0001**
Group: Stage	4	156	0.25	.91
Outcome variable: HF				
Intercept	1	4,110	183.36	<.0001
Age	1	38	0.64	.43
Gender	1	38	3.02	.09
Group	1	38	2.18	.15
Stage	5	4,110	170.80	**<.0001**
Group: Stage	5	4,110	9.51	**<.0001**
Outcome variable: SDNN				
Intercept	1	156	425.73	<.0001
Group (based on SE of 90%)	1	40	5.00	**.03**
Stage	4	156	9.19	**<.0001**
Group: Stage	4	156	0.12	.97

HF, high frequency heart rate variability (based on frequency bands from spectral analysis of HRV); HR, heart rate; SDNN, standard deviation of the R‐R interval.

*df*_n_ and *df*_d_ denote the numerator and denominator degrees of freedom, respectively. Model coefficients are in Table S1. *p* values below the significance threshold of .05 are in bold.

### 3.4 Heart rate variability

**FIGURE 5:**
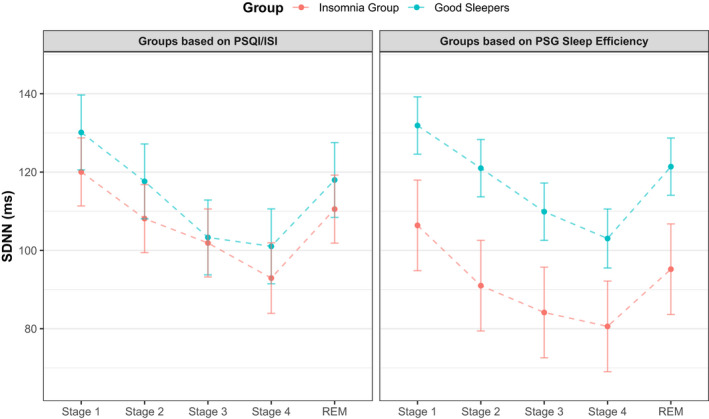
Model estimates of the standard deviation of the R–R interval (SDNN). Left: Group differences in SDNN across sleep stages, with groups defined by subjective sleep. A decline in SDNN from Stage 1 to 4 is observed. There is no discernible difference between groups. Right: Group differences in SDNN across sleep stages, with groups defined by objectively measured sleep (sleep efficiency > and <90%). As with the subjective groupings, a decline in SDNN is noticeable from Stage 1 to Stage 4. Values are mean ± *SE*

The mixed‐effects models highlighted no significant effect of group (*F*_40,1_ = 0.35, *p* = .56) on SDNN. The interaction between group and stage (*F*_156,4_ = 0.25, *p* = .91) was also not significant (Table 5; Figure 5; Top). There was a significant main effect of sleep stage on SDNN (*F*_156,4_ = 9.27, *p* < .0001). This is apparent in Figure 5, which shows a gradual decline in SDNN across sleep stages.

Sleep stage (*F*_42,047,5_ = 1762.9, *p* < .001) and the group × stage interaction were significant predictors of the HF ratio (Figure 4; bottom). Gender (*F*_38,1_ = 3.02, *p* = .09), age (*F*_38,1_ = 0.64, *p* = .42), and group (*F*_38,1_ = 2.18, *p* = .15) were not significant. Please refer to Tables S1 and S2 to review the full model output.

### 3.5 Insomnia with short sleep duration

Finally, we separated participants into groups based on their PSG sleep efficiency (of either > or <90%), to explore the differences in HRV linked to insomnia with short sleep duration. When groups were defined by sleep efficiency, we found a significant main effect of group (*F*_40,1_ = 5.00, *p* = .03) and stage (*F*_156,4_ = 9.19, *p* < .0001). The group × stage interaction (*F*_156,4_ = 0.12, *p* = .97) was not significant. Please refer to Table S2 to review the full model output.

## 4 DISCUSSION

The present study combined analysis of ambulatory sleep EEG with ECG in a sample of 43 university students (aged 18–30 years) to revisit the relationship between poor sleep and nocturnal HR and HRV. Importantly, the present study differs from previous studies in recruiting young, undiagnosed participants (reducing confounding of medication or comorbid medical disorders). Both groups were from the same population, matched for age and gender, and recordings were done in the participants’ homes. Arousal periods were removed from the calculation of HR and HRV to further remove any potential bias in the analysis.

We had four hypotheses. Our first hypothesis, that the insomnia group would exhibit poorer sleep‐onset latency and sleep efficiency than the good sleepers when observed in their own homes (as reported in earlier studies; Spiegelhalder et al., 2015), was partially supported by our present results. The insomnia group showed significantly higher wake after sleep onset and poorer sleep efficiency. Sleep‐onset latency was not significant when controlling for multiple comparisons.

The second hypothesis, that insomnia sufferers would display more arousals and a heightened arousal index across the night, was partially supported by our present analysis. A chi‐squared test highlighted that there was a significant between‐group difference in how arousals are distributed across the sleep stages. However, there was no significant difference in arousal index between the two groups. This might give a preliminary indication that the number of arousals across the stages of sleep (the highest being in Stage 2) may contribute to poorer subjective sleep quality (underpinned by physiological hyperarousal; Riemann et al., 2010).

Few investigations of arousals have been published that examine their distribution across stages. Of note, is the considerable amount of heterogeneity in the number of arousals between participants within each group. This suggests that arousals alone are unlikely to be decisive for subjective perception of sleep quality (Harvey & Tang, 2012), but it may relate to other neurophysiological underpinnings of sleep that are not accounted for in the present study.

### 4.1 HR and HRV in insomnia using time‐domain measures

With arousals excluded from HR time series, we anticipated increased nocturnal HR and decreased HRV during sleep for the insomnia group. Exploring between‐group differences, the poor sleepers had a significantly higher mean HR across Stages 2, 3, 4 and REM (Table 4; Figure 3). Figure 3 highlights there was substantial heterogeneity in mean HR, as in counts of arousals, among participants within each group. This again gives a preliminary indication that heightened HR is related to a complaint of poor sleep in the present study, which again would lend support to the hyperarousal theory of insomnia (Riemann et al., 2010).

To account for demographic variables and inter‐individual variability we fit linear mixed models to the HR data. We found a significant main effect of sleep stage (in line with previous research; Jarrin et al., 2016). There was no significant main effect of group, but there was a significant group‐by‐sleep stage interaction for nocturnal HR. This interaction highlighted the insomnia group to have a significantly lower wake–sleep reduction in their HR compared to the good sleeping group. This replicates and extends the results of Spiegelhalder et al., (2011), who reported a similar pattern in nocturnal HR when comparing patients with chronic insomnia to healthy controls. Their study was unable to include Stages 3 and 4 in their models because of the relative scarcity of deep sleep in the PSG recordings. In the present study, we have demonstrated that this blunted reduction in HR is also pronounced in slow‐wave sleep, in an attenuated unmedicated and undiagnosed student population with ambulatory PSG.

Our third hypothesis also specified that the insomnia group would show decreased HRV, but our present results only partially supported this. A summary measure of HRV, the SDNN, showed no significant differences between the groups. This is consistent with a recent critical review of HRV in patients with insomnia, which also produced negative findings (Dodds et al., 2016); and a more recent study by Eddie et al. (2020), which failed to find any significant differences in HR and HRV when comparing patients with major depressive disorder and insomnia to healthy controls (Eddie et al., 2020).

However, insomnia with short sleep duration (sleep efficiency <90%) did display an attenuated wake–sleep decrease in SDNN. This is consistent with the current literature, which purports insomnia with short sleep duration (<6 hr) to be the most “biologically aggressive” version of the disorder (Vgontzas et al., 2013). This is underscored by its association with increased cardiovascular autonomic dysfunction and risk for hypertension, alongside poorer cognitive functioning and treatment outcomes (Bathgate et al., 2017; Biddle et al., 2017; Jarrin et al., 2016).

### 4.2 HRV in insomnia using frequency‐domain measures

As with SDNN and nocturnal HR, HF was also linked to sleep stage (Figure 4), which presented as a significant main effect in our linear mixed model. The model further highlighted a significant stage × group interaction, which shows the insomnia group to have a higher HF during Wake. Figure 4 highlights the group difference is no longer apparent during sleep. This is partially in line with Eddie et al. (2020), who also found a significant main effect of sleep stage when predicting HF (across a major depressive disorder, an insomnia and a control group), but did not find a significant group × sleep stage interaction. The increased HF in the insomnia group during wake is contrary to our hypothesis, as higher HF during wake is thought to reflect greater resting vagal tone. Generally, this is considered as being more “adaptive” with regards to HRV, which would be expected to be seen in the good sleepers (Laborde et al., 2017). However, it is perhaps premature to interpret these findings further given this was not observed at any other stage and the insomnia group have significantly greater WASO and TIB (and therefore more Wake to analyse compared to the good sleepers).

### 4.3 Limitations

A number of caveats merit mention. The small sample size attenuates generalisability. The insomnia group scored significantly higher on anxiety and depression measures. These have been widely reported as highly comorbid disorders with insomnia, and have been thought of as having shared developmental roots (in dysregulated autonomic functioning; Eddie et al., 2020; Yang et al., 2011). However, this still adds bias to the sample. Furthermore, the comparability of the present study to others is limited given differences in how insomnia is defined and what controls/exclusion criteria are implemented.

## 5 CONCLUSIONS AND FUTURE DIRECTIONS

In summary, the poor sleepers displayed heightened WASO, poorer sleep efficiency, a greater arousal count and a significantly higher mean nocturnal HR across sleep Stages 2, 3, 4 and REM. The mixed‐effects models further highlighted a group × stage interaction for HR, indicating that the poor sleepers in the present study did show heightened physiological arousal during sleep. Insomnia with short sleep duration highlighted group differences in SDNN using linear mixed models. Taken together, these results lend credence to the hyperarousal model of insomnia and may help to explain the increased cardiovascular morbidity and mortality reported in patients with insomnia. Future research with larger sample sizes, also employing unmedicated populations with ambulatory PSG, should aim to replicate these results, to add clarity to some of the limitations of the current literature to date.

## CONFLICT OF INTEREST

J.C., J.P., R.H., D.S., K.W. declare no conflict of interest. R.G.F. has held consulting contracts with Dyson Ltd and is one of the academic founders and directors of the Oxford spin‐off Circadian Therapeutics Ltd since 2016.

## AUTHOR CONTRIBUTIONS

JC: Conceptualisation, investigation, formal analysis, visualisation, writing (original draft), supervision. JP: Formal analysis. RH: Methodology, software, validation. RGF: Funding acquisition, resources. DS: Conceptualisation, methodology, validation, supervision. KW: Conceptualisation, writing (review and editing), resources, funding acquisition, supervision.

## Supporting information

Supplementary Material

## Data Availability

Data available on request from the authors. The data that support the findings of this study are available from the corresponding author upon reasonable request.
