# Supplementary material for: Revisiting nocturnal heart rate and heart rate variability in insomnia: A polysomnography‐based comparison of young self‐reported good and poor sleepers
Source: J Sleep Res. 2021 Feb 23;30(4):e13278. doi: 10.1111/jsr.13278 (PMC8577225; doi:10.1111/jsr.13278)
Supplement: Supplementary file 1 — Supplementary Material [file JSR-30-e13278-s001.docx]

**Supplementary Materials :**

**Table 1 Linear Mixed Effects Model Output with HR and SDNN as Outcome Variables and a Random effect of Subject ID**

|  | Value | Std. Error | df | | t |
| --- | --- | --- | --- | --- | --- |
| Outcome Variable: HR | | | |  |  |
| Intercept | 50.04 | 8.57 | 42047 | | 5.84 |
| Age | 0.25 | 0.37 | 38 | | 0.69 |
| Woman | 6.98 | 2.48 | 38 | | 0.80 |
| Insomnia Group | 1.96 | 2.45 | 38 | | 0.80 |
| Stage 1 | -0.91 | 0.32 | 42047 | | -2.84 |
| Stage 2 | -2.56 | 0.15 | 42047 | | -16.86 |
| Stage 3 | -2.40 | 0.28 | 42047 | | -8.49 |
| Stage 4 | -1.14 | 0.20 | 42047 | | -5.80 |
| Wake | 12.94 | 0.19 | 42047 | | 67.65 |
| Insomnia Group: Stage 1 | -1.78 | 0.44 | 42047 | | -4.07 |
| Insomnia Group: Stage 2 | -0.10 | 0.21 | 42047 | | -0.51 |
| Insomnia Group: Stage 3 | 1.24 | 0.37 | 42047 | | 3.31 |
| Insomnia Group: Stage 4 | 1.32 | 0.27 | 42047 | | 4.95 |
| Insomnia Group: Wake | -3.04 | 0.25 | 42047 | | -11.98 |
| Outcome Variable: SDNN |  |  |  | |  |
| Intercept | 117.98 | 9.56 | 156 | | 12.34 |
| Insomnia Group | -7.43 | 12.91 | 40 | | -0.58 |
| Stage 1 | 12.15 | 7.30 | 156 | | 1.66 |
| Stage 2 | -0.35 | 7.30 | 156 | | -0.48 |
| Stage 3 | -14.67 | 7.30 | 156 | | -2.01 |
| Stage 4 | -16.94 | 7.30 | 156 | | -2.32 |
| Insomnia Group: Stage 1 | -2.65 | 9.87 | 156 | | -0.27 |
| Insomnia Group: Stage 2 | -2.07 | 9.87 | 156 | | -0.21 |
| Insomnia Group: Stage 3 | 6.02 | 9.87 | 156 | | 0.61 |
| Insomnia Group: Stage 4 | -0.67 | 10.15 | 156 | | -0.07 |

**Table 2 Linear Mixed Effects Model Output with HF and SDNN as Outcome Variables and a Random effect of Subject ID**

|  | Value | Std. Error | df | | t |
| --- | --- | --- | --- | --- | --- |
| Outcome Variable: HF | | | |  |  |
| Intercept | 646.67 | 346.73 | 4110 | | 1.87 |
| Age | -6.03 | 14.86 | 38 | | -0.41 |
| Woman | -148.35 | 99.77 | 38 | | -1.49 |
| Insomnia Group | 16.39 | 108.84 | 38 | | 0.15 |
| Stage 1 | -30.70 | 103.66 | 4110 | | -0.30 |
| Stage 2 | 26.62 | 41.56 | 4110 | | 0.64 |
| Stage 3 | 3.06 | 88.89 | 4110 | | 0.03 |
| Stage 4 | -2.47 | 48.45 | 4110 | | -0.05 |
| Wake | 334.86 | 39.40 | 4110 | | 8.50 |
| Insomnia Group: Stage 1 | -14.55 | 150.66 | 4110 | | -0.10 |
| Insomnia Group: Stage 2 | 1.46 | 56.95 | 4110 | | 0.03 |
| Insomnia Group: Stage 3 | 10.06 | 116.94 | 4110 | | 0.09 |
| Insomnia Group: Stage 4 | -33.80 | 66.17 | 4110 | | -0.51 |
| Insomnia Group: Wake | 203.44 | 53.76 | 4110 | | 3.78 |
| Outcome Variable: SDNN |  |  |  | |  |
| Intercept | 121.39 | 7.32 | 156 | | 16.59 |
| Insomnia Group (based on SE of 90%) | -26.18 | 13.69 | 40 | | -1.91 |
| Stage 1 | 10.50 | 5.82 | 156 | | 1.80 |
| Stage 2 | -0.39 | 5.82 | 156 | | -0.07 |
| Stage 3 | -11.50 | 5.82 | 156 | | -1.98 |
| Stage 4 | -18.35 | 6.10 | 156 | | -3.01 |
| Insomnia Group: Stage 1 | 0.69 | 10.89 | 156 | | 0.06 |
| Insomnia Group: Stage 2 | -3.81 | 10.89 | 156 | | -0.35 |
| Insomnia Group: Stage 3 | 0.45 | 10.89 | 156 | | 0.04 |
| Insomnia Group: Stage 4 | 3.74 | 11.04 | 156 | | 0.34 |
